# Supplementary material for: Discovery and characterization of potent pan-variant SARS-CoV-2 neutralizing antibodies from individuals with Omicron breakthrough infection
Source: Nat Commun. 2023 Jun 15;14:3537. doi: 10.1038/s41467-023-39267-x (PMC10267556; doi:10.1038/s41467-023-39267-x)
Supplement: Supplementary file 3 — Reporting Summary [file 41467_2023_39267_MOESM3_ESM.pdf]

nature portfolio

Corresponding author(s): Yu Guo, Zihe Rao, Tao Cheng, Ximo Wang, Ping Zhu, Fengjiang Liu, Dongsheng Xiong, Xinwen Chen, Xiaoming Yang, Qi Yang

Last updated by author(s): Apr 7, 2023

Reporting Summary

Nature Portfolio wishes to improve the reproducibility of the work that we publish. This form provides structure for consistency and transparency in reporting. For further information on Nature Portfolio policies, see our Editorial Policies and the Editorial Policy Checklist.

Statistics

For all statistical analyses, confirm that the following items are present in the figure legend, table legend, main text, or Methods section.

n/a	Confirmed	
☐	☒	The exact sample size (*n*) for each experimental group/condition, given as a discrete number and unit of measurement
☐	☒	A statement on whether measurements were taken from distinct samples or whether the same sample was measured repeatedly
☐	☒	The statistical test(s) used AND whether they are one- or two-sided<br>*Only common tests should be described solely by name; describe more complex techniques in the Methods section.*
☒	☐	A description of all covariates tested
☒	☐	A description of any assumptions or corrections, such as tests of normality and adjustment for multiple comparisons
☐	☒	A full description of the statistical parameters including central tendency (e.g. means) or other basic estimates (e.g. regression coefficient) AND variation (e.g. standard deviation) or associated estimates of uncertainty (e.g. confidence intervals)
☐	☒	For null hypothesis testing, the test statistic (e.g. *F*, *t*, *r*) with confidence intervals, effect sizes, degrees of freedom and *P* value noted<br>*Give P values as exact values whenever suitable.*
☒	☐	For Bayesian analysis, information on the choice of priors and Markov chain Monte Carlo settings
☒	☐	For hierarchical and complex designs, identification of the appropriate level for tests and full reporting of outcomes
☒	☐	Estimates of effect sizes (e.g. Cohen's *d*, Pearson's *r*), indicating how they were calculated

Our web collection on statistics for biologists contains articles on many of the points above.

Software and code

Policy information about availability of computer code

Data collection	CPE was evaluated using a Celigo Image Cytometer (Celigo).<br>Single-cell data was obtained by 10x Chromium Next GEM Chip K and sequenced using an Illumina platform Novaseq6000.<br>SPR data was obtained by Biacore Insight Evaluation Software (Biacore 8K, Cytiva).<br>Neutralization data was obtained by GraphPad Prism.<br>Cryo-EM data collection was performed using either a Titan Krios G3 equipped with a K3 direct detection camera, and MotionCorr2.
Data analysis	Data representation and statistical analysis were performed using GraphPad Prism 6.0 and ggplot2.<br>Single-cell expression matrix was generated by Cellranger (v6.1.2).<br>QC was achieved by Seurat (v4.0.3).<br>Doublets were evaluated by Scrublet (v0.2.1).<br>Integration and clustering were performed by scanpy (v1.5.1).<br>BCR contig sequences were assembled by Cellranger (v6.1.2).<br>Clonotypes were obtained by Python scripts.<br>Mismatches and gaps were obtained by Igblast (v1.18.0).<br>Cryo-EM data processing was carried out using cryoSPARC (v2.15.0) and UCSF ChimeraX (v1.2.5). Coot (v0.8.9.2) and Phenix (v1.16) were used for cryo-EM structural modeling and refinement. Structure figures were prepared using UCSF Chimera (v1.16) and Pymol (v2.2.0).

For manuscripts utilizing custom algorithms or software that are central to the research but not yet described in published literature, software must be made available to editors and reviewers. We strongly encourage code deposition in a community repository (e.g. GitHub). See the Nature Portfolio guidelines for submitting code & software for further information.

Data

Policy information about availability of data

All manuscripts must include a data availability statement. This statement should provide the following information, where applicable:

- Accession codes, unique identifiers, or web links for publicly available datasets
- A description of any restrictions on data availability
- For clinical datasets or third party data, please ensure that the statement adheres to our policy

All data that support the findings of this study have been deposited in the paper and Supplementary materials
Cryo-EM density maps have been deposited in the Electron Microscopy Data Bank with accession codes EMD-34181, EMD-34124, EMD-34125, EMD-34126, EMD-34127, EMD-34128, EMD-34129, EMD-34130, EMD-34131, EMD-34132, EMD-34133, EMD-34134 and EMD-34135, respectively. Structure coordinates have been deposited in the Protein Data Bank with accession codes with accession codes 8GOU, 7YVE, 7YVF, 7YVG, 7YVH, 7YVI, 7YVJ,7YVK,7YVL, 7YVM, 7YVN, 7YVO and 7YVP, respectively. The atomic models generated from X-ray crystallographic has been deposited at the Protein Data Bank under accession codes PDB 8GPY. All single-cell sequencing data has been deposited at National Genomics Data Center (https://ngdc.cncb.ac.cn/) with access number HRA003208. The access to the single-cell sequencing data is restricted to avoid commercial or improper use, which could be shared for any research after simple email correspondence. Source data are provided with this paper. The codes used in this study are available from the corresponding authors upon reasonable request.

Human research participants

Policy information about studies involving human research participants and Sex and Gender in Research.

Reporting on sex and gender	The unvaccinated persons: 2 males and 2 females with an average age of 58, 1 dose-vaccinated persons:1 females with an age of 42, 2 dose-vaccinated persons: 8 males and 5 females with an average age of 39, 3 dose-vaccinated persons: 4 males and 9 females with an average age of 44, the vaccinated information unknown persons: 4 males and 3 females with an average age of 45. We did not perform sex- and gender-based analyses, antibody response shouldnot be affected by sex.
Population characteristics	38 Omicron patients in infection stage from Tianjin Hai he Hospital were involved in this study, 26 of them has received two or three doses of BBBIP-CorV inactivated SARS-CoV-2 vaccine before BA.1 infection, 1 of them has received one doses of BBBIP-CorV inactivated SARS-CoV-2 vaccine before BA.1 infection, 4 of them has not been vaccinated before BA.1 infection, and 7 of them vaccinated information unknown. All donors symptoms were mild or moderate.
Recruitment	All donors gave informed consent for the provision of specimens for testing, further diagnosis, and scientific research during hospitalization. The requirement for participants to be recovered patients after infection with BA.1. The only exclusion criteria used were HIV or other debilitating disease.
Ethics oversight	This study was approved by the Ethics Committee from Haihe Laboratory of Cell Ecosystem (ethical approval number HHL2022005-EC-1).Written informed consents were obtained from each enrolled patient in accordance with the Declaration of Helsinki.

Note that full information on the approval of the study protocol must also be provided in the manuscript.

Field-specific reporting

Please select the one below that is the best fit for your research. If you are not sure, read the appropriate sections before making your selection.

☒ Life sciences ☐ Behavioural & social sciences ☐ Ecological, evolutionary & environmental sciences

For a reference copy of the document with all sections, see nature.com/documents/nr-reporting-summary-flat.pdf

Life sciences study design

All studies must disclose on these points even when the disclosure is negative.

Sample size	For human studies, sample size was not applicable because no sample size calculation was performed during the study design. Other studies, sample sizes are already indicated in the figures and legend to ensure valid statistical power and good technical reproducibility.
Data exclusions	No data was excluded.
Replication	For live virus and pseudotype virus neutralization, ELISA, SPR experiments, at least three independent experiments were successfully performed.
Randomization	For human studies, except donor1, dornor2 and donor3, the remaining donors of 38 were randomly divided into three groups (bulk1, bulk2 and bulk3). Other studies, Randomization is not required in this study.
Blinding	The study was not blinded. The key experiments were repeated independently by different investigators in the study.

Reporting for specific materials, systems and methods

We require information from authors about some types of materials, experimental systems and methods used in many studies. Here, indicate whether each material, system or method listed is relevant to your study. If you are not sure if a list item applies to your research, read the appropriate section before selecting a response.

Materials & experimental systems

n/a	Involved in the study
☐	☒ Antibodies
☐	☒ Eukaryotic cell lines
☒	☐ Palaeontology and archaeology
☐	☒ Animals and other organisms
☒	☐ Clinical data
☒	☐ Dual use research of concern

Methods

n/a	Involved in the study
☒	☐ ChIP-seq
☒	☐ Flow cytometry
☒	☐ MRI-based neuroimaging

Antibodies

Antibodies used

For RBD-binding B cells enrichment: B cell negative selection kit (STEMCELL, #19554)
ELISA for binding assay: anti-human IgG (H+L)/HRP (JACKSON, #109-035-098)
ELISA for blocking assay:Anti-his-HRP (Sino Biological Inc, #A5327)
ELISA for competing assay:Anti-Avi-Tag Monoclonal Antibody (5G11), HRP Conjugated (Abbkine, Cat #: A02210HRP)
286 pairs of heavy- and light-chain plasmids were mixed with transfection reagent TF02 (Sinofection, #STF02) respectively and added into HEK293 cells. HEK293 cells were cultured in SMS 293-SUPI medium (Sinofection, #M293-SUPI-100) at at 37 degree, with 5% CO2. Seven days after transtection, partial condition medium were taken for binding identification by ELISA. Ten days after transfection, the cultured medium were harvested and mAbs were purified using Protein A column (GE, Hitrap Protein A HP). Antibodies use papain to generate Fab fragments. Briefly, antibodies were first cleaved by papain for 8h at 37°C. The mixture then attached Fc crystallizable fragments through Protein A columns, allowing Fab to flow out. The Fab was collected and dialyzed into PBS.

Validation

All antibodies were expressed using HEK293F cell lines with codon- optimized cDNA and human IgGI constant regions. All antibodies' species and specificity to RBD among variants were validated by ELISA. Details and sequences for all SARS-CoV-2 antibodies evaluated in this study is included in Supplementary materials.

Eukaryotic cell lines

Policy information about cell lines and Sex and Gender in Research

Cell line source(s)

Vero E6 cells were purchased from ATCC (cat. CRL-1586).
Sf9 cells were purchased from ATCC (cat. CRL-1711).

Authentication

No authentication was performed beyond manufacturer standards.

Mycoplasma contamination

We confirm that all cells were tested as mycoplasma negative.

Commonly misidentified lines (See ICLAC register)

No commonly misidentified cell lines were used.

Animals and other research organisms

Policy information about studies involving animals; ARRIVE guidelines recommended for reporting animal research, and Sex and Gender in Research

Laboratory animals

K18-hACE2 transgenic mice

Wild animals

No wild animals

Reporting on sex

Female

Field-collected samples

Forty-eight female hACE2 transgenic mice were divided into eight groups with six mice in each group to evaluate the efficacy of cocktail TH027+TH132 in prophylaxis and therapy. For the prophylactic treatment group, TH027 and TH132 or cocktail TH027+TH132 were administered at 2 h before virus challenge. For the therapeutic treatment group, cocktail TH027+TH132 administration was delayed until 2 h.p.i.. K18-hACE2 transgenic mice received an intraperitoneal dose of 5 or 20 mg/kg in a volume of 100 µL. An equivalent volume of PBS was administered as control. On the day of infection, the hACE2 mice were intranasally inoculated with either 1×10^4 TCID50 Omicron BA.5, pre-diluted in 50 µL DMEM. Mice were killed at the designated timepoints and organ tissues were sampled for virological and histopathological analyses.

Ethics oversight	The use of K18-hACE2 transgenic mice has received ethical approval from the Animal Ethics Committee at Guangzhou Customs Inspection and Quarantine Technology Center (IQTC20221003).

Note that full information on the approval of the study protocol must also be provided in the manuscript.
