# Supplementary material for: Discovery and characterization of potent pan-variant SARS-CoV-2 neutralizing antibodies from individuals with Omicron breakthrough infection
Source: Nat Commun. 2023 Jun 15;14:3537. doi: 10.1038/s41467-023-39267-x (PMC10267556; doi:10.1038/s41467-023-39267-x)
Supplement: Supplementary file 4 — Supplementary Information [file 41467_2023_39267_MOESM4_ESM.pdf]

Supplementary information

Discovery and characterization of potent pan-variant SARS-CoV-2 neutralizing antibodies from individuals with Omicron breakthrough infection

Yu Guo[1,2,3, 4,*,†] , Guangshun Zhang[1,3,5,6,*], Qi Yang[3,*,†], Xiaowei Xie[2,*], Yang Lu[2,*], Xuelian Cheng[2,*], Hui Wang[4,5,*], Jingxi Liang[1,7,*], Jielin Tang[3,*], Yuxin Gao[1,5,6,*], Hang Shang[1,5,6], Jun Dai[9], Yongxia Shi[9], Jiaxi Zhou[2], Jun Zhou[1,2], Hangtian Guo[7], Haitao Yang[7], Jianwei Qi[2], Lijun Liu[2], Shihui Ma[2], Biao Zhang[2], Qianyu Huo[2], Yi Xie[6], Junping Wu[8], Fang Dong[1,6], Song Zhang[1,6], Zhiyong Lou[3], Yan Gao[7], Zidan Song[1,3,5,6], Wenming Wang[1,5,6], Zixian Sun[3], Xiaoming Yang[4,5,†], Dongsheng Xiong[2,†], Fengjiang Liu[3,†], Xinwen Chen[3,†], Ping Zhu[2,†], Ximo Wang[8,†], Tao Cheng[2,†], Zihe Rao[1,3,5,7,†]

Correspondence to: guoyu@nankai.edu.cn

Supplementary Figures S1-S13
Supplementary Tables S1-S7

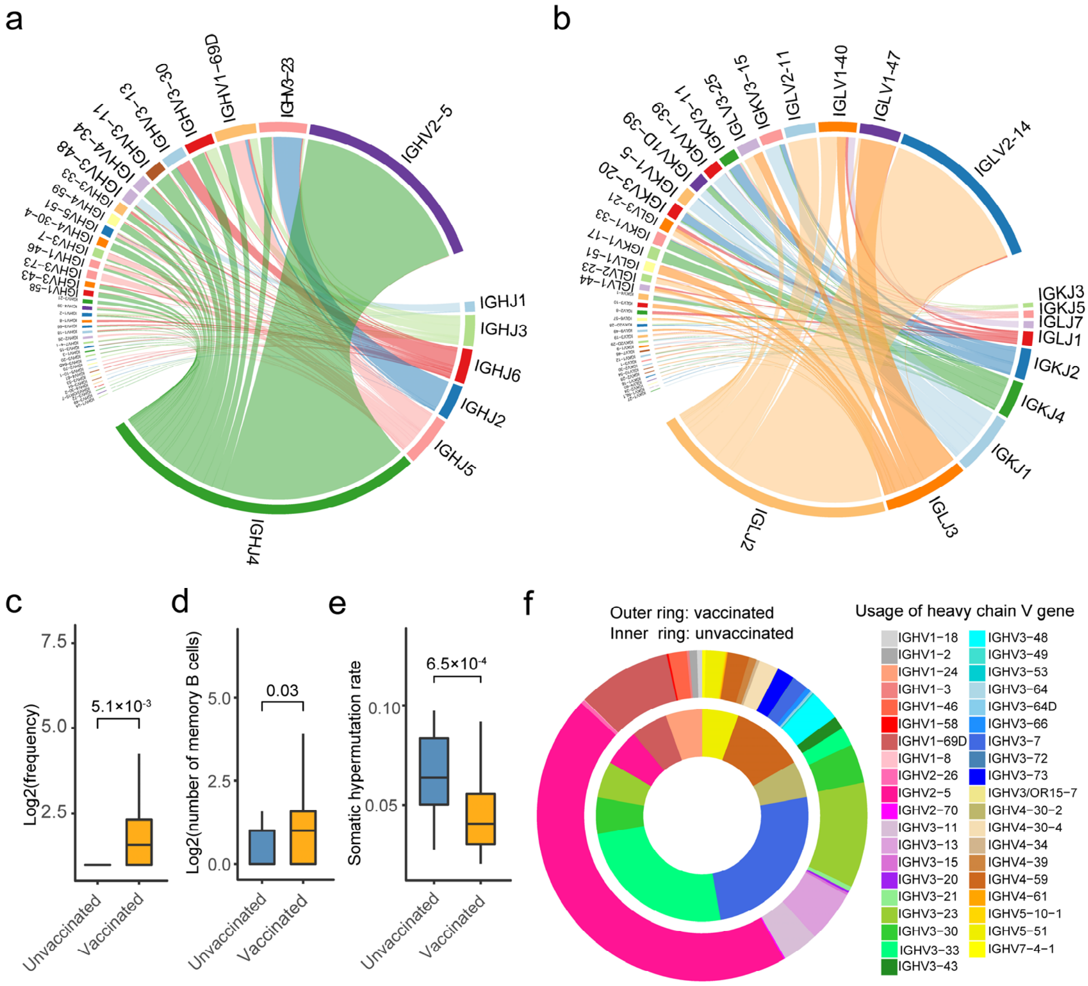

Fig. S1. Unvaccinated versus vaccinated.

(a) Circos plot showing the VDJ gene rearrangements of the heavy chain for 286 high-confidence candidates.

(b) Circos plot showing the VDJ gene rearrangements of the light chain for 286 high-confidence candidates.

(c) Box plot showing the frequency between unvaccinated and vaccinated Omicron patients. Two-sided Wilcoxon test (clonotypes from unvaccinated patients: n=16, clonotypes from vaccinated patients: n=259).

(d) Box plot showing the number of memory B cells between unvaccinated and vaccinated Omicron patients. Two-sided Wilcoxon test (clonotypes from unvaccinated patients: n=16, clonotypes from vaccinated patients: n=259).

(e) Box plot showing the somatic hypermutation rate between unvaccinated and vaccinated Omicron patients. Two-sided Wilcoxon test (clonotypes from unvaccinated patients: n=16, clonotypes from vaccinated patients: n=259).

(f) Pie plot displaying the usage percentage of each heavy chain V gene between unvaccinated (inner circle) and vaccinated (outer circle) Omicron patients.

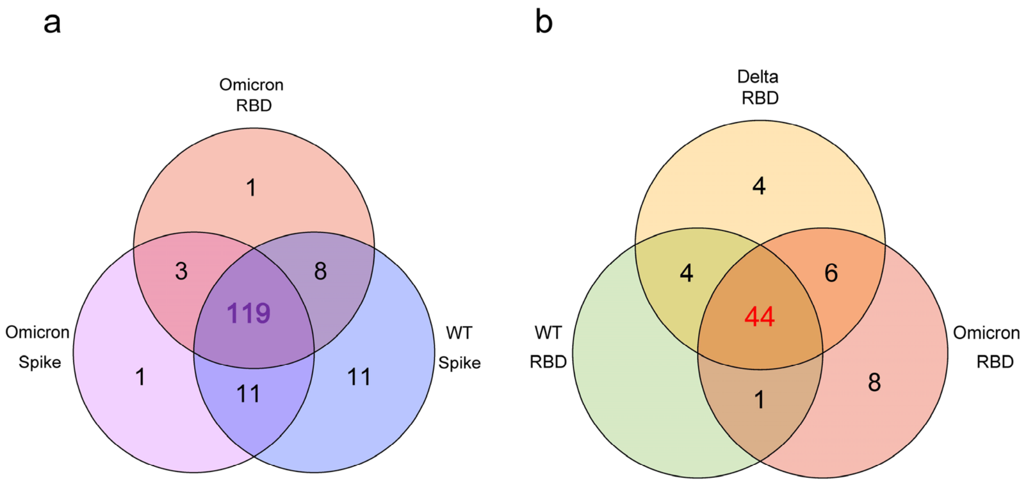

Fig. S2. Cross-reaction profile of selected antibodies.
(a) Cross-affinity ability of positive binding mAbs (OD450>4). A total of 119 broad-spectrum binding mAbs were chosen for the next tests. A total of 146 mAbs could not bind any of the variants, which was not shown in the Venn graph.
(b) Cross-competition ability of positive blocking mAbs (IC50<25 μg/ml). Forty-four broad-spectrum blocking mAbs were chosen for further study. A total of 52 mAbs could not block any of the variants, which was not shown in the Venn graph.

a

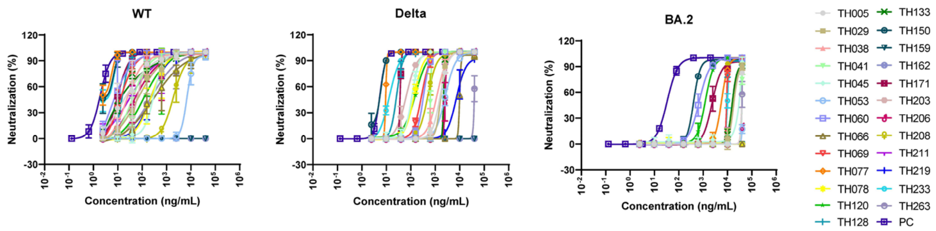

b

NT_{50} (ng/mL)	TH005	TH029	TH038	TH041	TH045	TH053	TH060	TH066	TH069	TH077	TH078	TH120	TH128
WT	14.52	68.28	23.50	42.39	306.3	6911	5.236	303.3	12.35	5.548	245.9	118.6	15.54
Delta	1618	1713	1183	636.3	114.1	2615	334.4	9949	277.5	9.489	136.5	156.3	26.37
BA.2	57483	17984	58542	57507	111313	59747	694.3	/	9263	5724	7750	1251	9219
NT_{50} (ng/mL)	TH133	TH150	TH159	TH162	TH171	TH203	TH206	TH208	TH211	TH219	TH233	TH263	PC
WT	20.45	6.649	/	/	9.438	65.02	44.52	1914	30.19	237.7	6.956	235.8	1.911
Delta	2195	6.027	/	/	36.59	65.02	2554	497.3	2211	7302	13.34	58506	5.457
BA.2	15514	407.3	/	/	2336	17436	58043	/	16612	/	9955	58506	33.66

Fig. S3. Neutralization potency of individual monoclonal antibodies from recipients of breakthrough infections against 3 SARS-CoV-2 strains.
(a) Neutralization curves for 25 selected antibodies toward WT virus, Delta and Omicron BA.2 strains. Serial dilutions of monoclonal antibodies were incubated with SARS-CoV-2 (MOI=0.01) for 1 h at 37 °C and then added to Vero E6 cells. CPE was measured 48 hours post-infection as a read-out for virus infectivity. PC as a positive control. Data are graphed as percent neutralization relative to the virus-only infection control. (elite antibodies: n=4 technical replicates, PC: n=3 technical replicates). Data are presented as mean values ± SD.
(b) Data are graphed as percent neutralization relative to the virus-only infection control. PC as a positive control. All experiments were performed in duplicate.

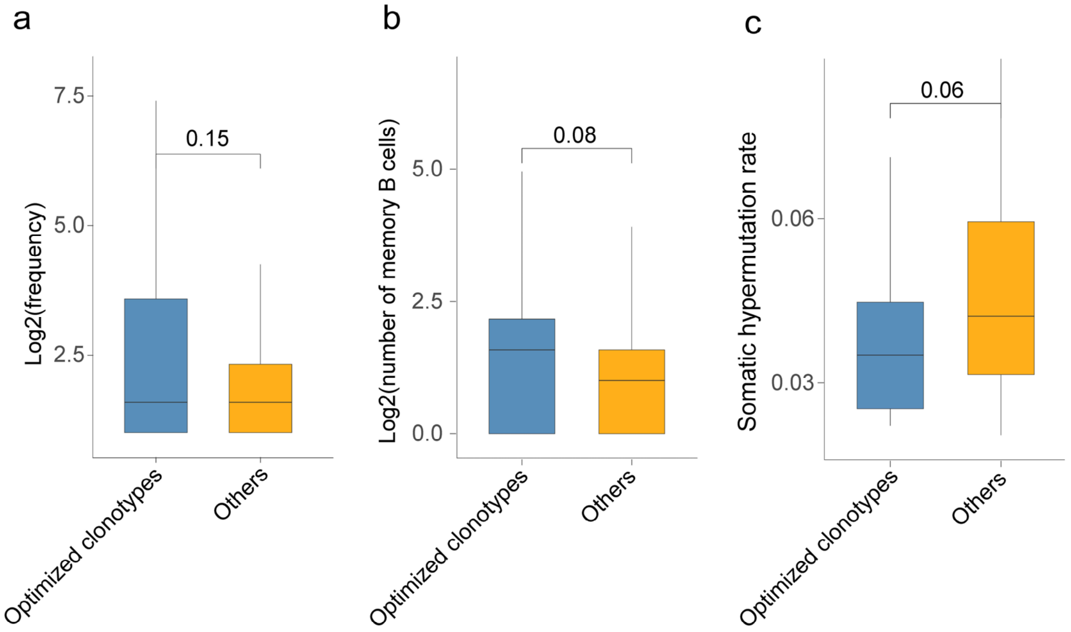

Fig. S4. Characteristics of 19 optimized clonotypes. Box plots showing the differences between optimized clonotypes and the remaining clonotypes for frequency (a), the number of memory B cells (b) and somatic hypermutation rate (c). Two-sided Wilcoxon test (optimized clonotypes: n=19, the remaining clonotypes: n=267).

a

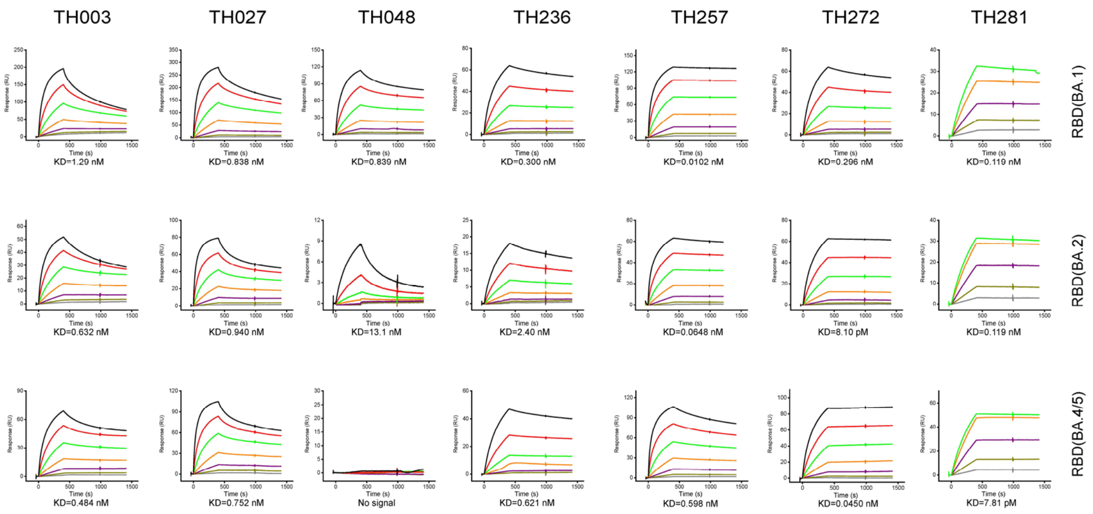

Fig. S5. The affinity of 7 candidate antibodies to different Omicron sublineages was detected by SPR.

(a) Threefold serial dilutions from 50 nM to 0.068 nM. Kinetic data from one representative experiment were fit to a 1:1 binding model by Biacore 8K. No signal means not detected.

	TH003	TH004	TH015	TH025	TH027	TH051	TH089	TH095	TH111	TH183	TH236	TH257	TH272	TH273	TH274	TH048	TH132	TH139	TH281
TH003	92	58	66	69	59	41	79	98	87	88	108	108	107	95	96	-25	-24	-2	-1
TH004	21	89	18	18	13	22	29	33	24	21	52	53	61	33	23	-7	-4	2	-4
TH015	63	71	93	69	70	74	83	95	92	81	100	100	101	94	91	-40	-125	-100	-20
TH025	56	39	47	76	44	53	65	79	72	75	95	96	96	79	78	-85	-74	-47	-5
TH027	74	57	76	74	83	77	81	90	87	88	95	95	92	89	79	-5	-86	-35	-12
TH051	59	33	35	41	39	78	71	86	79	79	90	90	90	84	82	-71	-126	-60	-29
TH089	65	56	57	59	56	67	81	81	77	79	83	81	82	80	78	-38	-72	-26	-5
TH095	41	40	37	35	39	39	46	75	75	70	98	102	99	65	64	-29	-48	-17	-6
TH111	64	64	70	69	69	66	65	85	88	71	103	102	102	76	77	-40	-77	-23	-16
TH183	65	67	62	66	64	70	75	86	80	85	101	101	102	83	85	11	-6	-1	-1
TH236	33	37	58	52	40	25	34	48	36	34	92	106	86	34	48	-23	-42	-27	-2
TH257	47	35	69	62	61	43	49	61	39	47	90	115	81	47	46	12	-25	-3	-8
TH272	63	57	74	70	67	58	64	77	61	61	103	115	96	62	65	-2	-2	-2	3
TH273	83	65	85	82	82	91	91	89	89	56	97	99	95	86	89	-3	-36	-34	-31
TH274	92	79	87	53	66	89	96	100	99	75	81	95	104	96	100	54	-1	28	0
TH048	-37	-63	-66	-71	-48	-46	-25	-46	-74	-61	-66	-28	-33	-50	-64	75	77	98	91
TH132	4	-11	-14	-7	-7	6	18	8	-20	3	-13	-15	14	-3	2	26	89	90	94
TH139	-11	-16	-35	-33	-28	-15	0	-7	-22	-14	-16	-27	-3	-16	-9	20	86	95	106
TH281	60	65	61	65	60	53	68	69	55	57	22	34	50	53	66	50	91	101	108

Fig. S6. Epitope binning by competitive ELISA. The mullions are coated antibodies, and the horizontal are competing antibodies. Two groups were identified (orange and green).

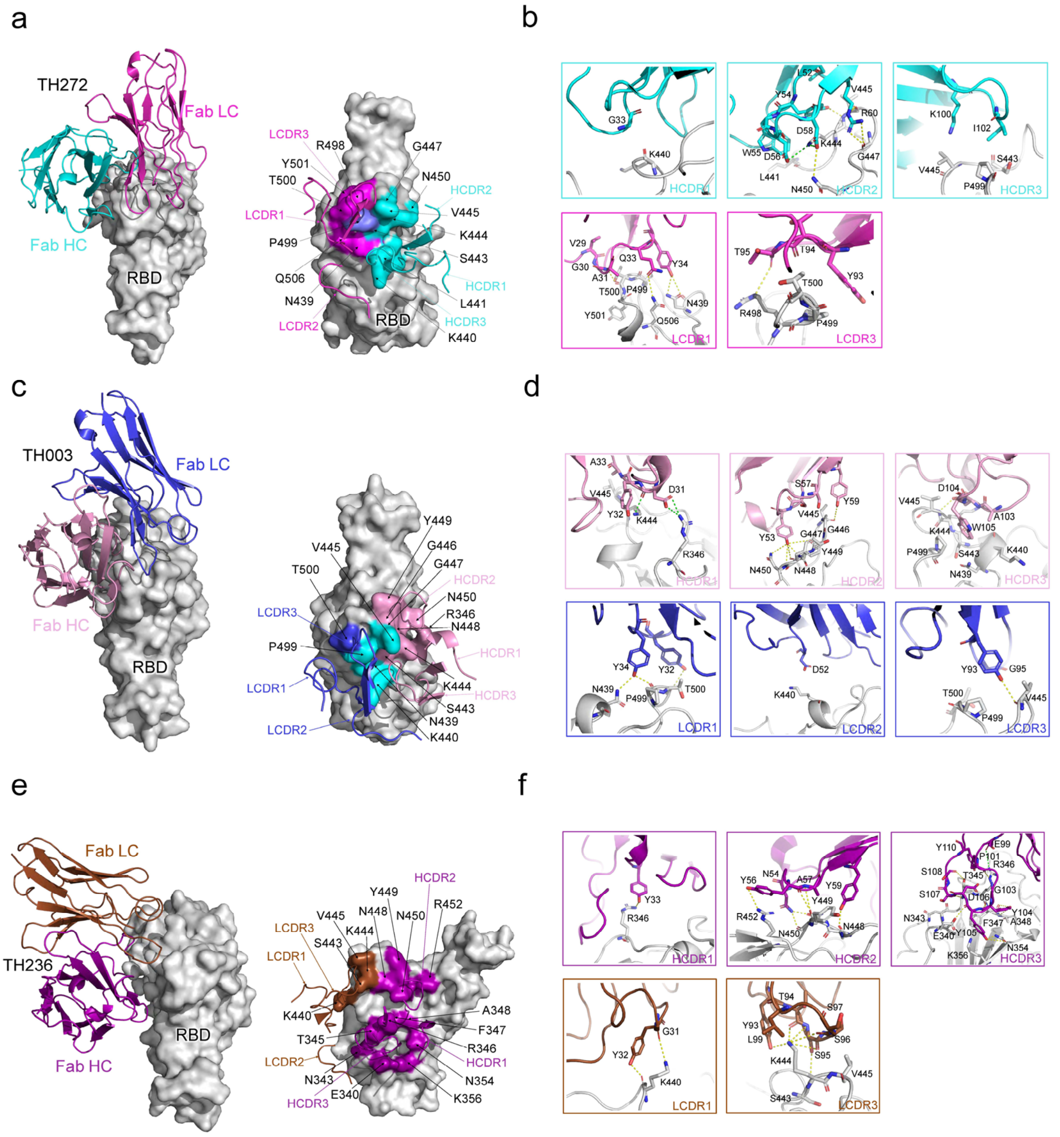

Fig. S7. Structural analysis of TH272, TH003, TH236 and the BA.4/5 RBD complex.
(a) The overall TH272-RBD complex structure superimposed with the BA.4/5 RBD. The TH272 heavy chain (colored cyan) and light chain (colored magenta) are displayed in cartoon representation. The BA.4/5 RBD is colored in gray and displayed in surface representation. The epitope of TH027 is shown in surface representation. The CDR loops of heavy chain (HCDR) and light chain (LCDR) are colored in cyans and magenta, respectively. The epitopes from the heavy chain and light chain are colored in cyan and magenta, respectively. P499, which contacts both the heavy chain and light chain, is colored blue.
(b) The residue interactions between the SARS-CoV-2 RBD and the HCDR and LCDR of TH272. Hydrogen bond interactions are shown as yellow dashed lines. Salt bridge interactions are shown as green dashed lines. The residues are shown in sticks with identical colors to (a).

(c) The overall TH003-RBD complex structure superimposed with the BA.4/5 RBD. The TH003 heavy chain (colored pink) and light chain (colored blue) are displayed in cartoon representation. The BA.4/5 RBD is colored in gray and displayed in surface representation. The epitope of TH003 is shown in surface representation. The CDR loops of the heavy chain (HCDR) and light chain (LCDR) are colored pink and blue, respectively. The epitopes from the heavy chain and light chain are colored pink and blue, respectively. N439, K440, V445 and P499, which contact both the heavy chain and light chain, are colored in cyans.
(d) The residue interactions between the SARS-CoV-2 RBD and the HCDR and LCDR of TH003. Hydrogen bond interactions are shown as yellow dashed lines. Salt bridge interactions are shown as green dashed lines. The residues are shown in sticks with identical colors to (c).
(e) The overall TH236-RBD complex structure superimposed with the BA.4/5 RBD. The TH236 heavy chain (colored purple) and light chain (colored brown) are displayed in cartoon representation. The BA.4/5 RBD is colored in gray and displayed in surface representation. The epitope of TH236 is shown in surface representation. The CDR loops of the heavy chain (HCDR) and light chain (LCDR) are colored purple and brown, respectively. The epitopes from the heavy chain and light chain are colored purple and brown, respectively.
(f) The residue interactions between the SARS-CoV-2 RBD and the HCDR and LCDR of TH236. Hydrogen bond interactions are shown as yellow dashed lines. Salt bridge interactions are shown as green dashed lines. The residues are shown in sticks with identical colors to (e).

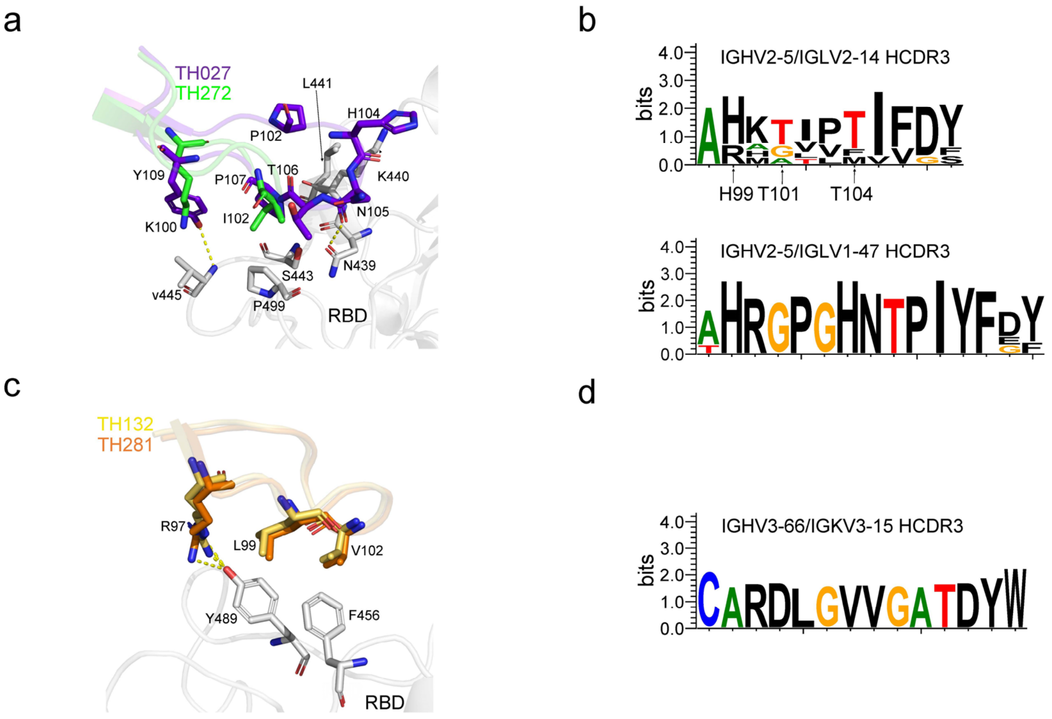

Fig. S8. HCDR3 structure alignment of IGHV2-5 or IGHV3-66/53.

(a) The HCDR3 structure of TH027 or TH272 is displayed in cartoon representation. The residues are shown in sticks. RBD is colored in gray. The color scheme is the same as in Fig. 4a.

(b) Amino acid conservation analysis of the HCDR3 sequence between IGHV2-5/IGLV2-14 (top) and IGHV2-5/IGLV1-47 (bottom) by WebLogo 3.

(c) The HCDR3 structure of TH132 or TH281 is displayed in cartoon representation. The residues are shown in sticks. RBD is colored in gray. The color scheme is the same as in Fig. 4a.

(d) Amino acid conservation analysis of the HCDR3 sequence between IGHV3-66/IGKV3-15 by WebLogo 3.

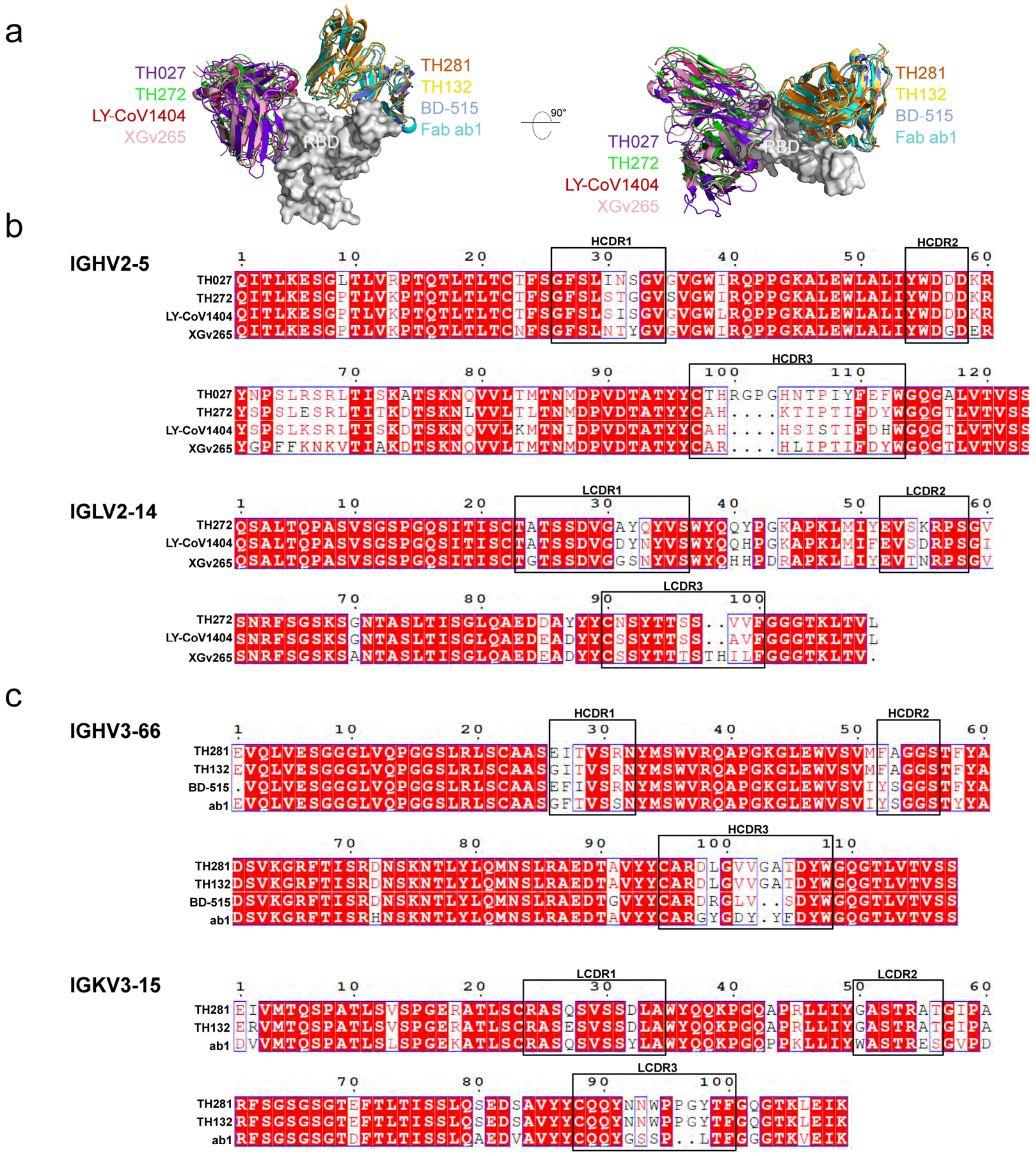

Fig. S9. Structure and sequence alignment of TH027 or TH281 to other reported antibodies from the IGHV2-5 or 3-66 germline.

(a) Superposition of TH027 (deep blue), TH272 (green), LY-CoV1404 (deep red, PDB 7MMO), XGv265 (pink, PDB 7WEE), TH281 (orange), TH132 (yellow), BD-515 (light blue, PDB 7E88), and ab1 (cyans, PDB 7MJJ). RBD is colored in gray.

(b) Alignment of the heavy and light chain variable domain sequences of TH027 and TH272 with LY-CoV14104 and XGv265.

(c) Alignment of the heavy and light chain variable domain sequences of TH281 and TH132 with BD-515 and ab1.

a

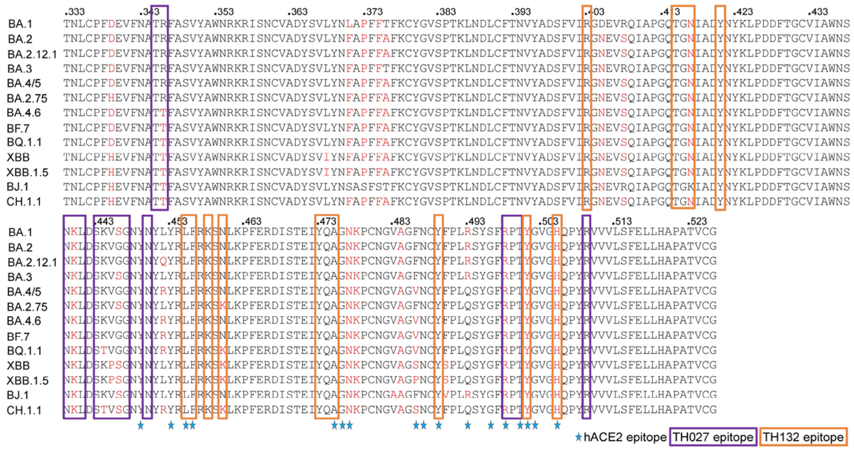

b

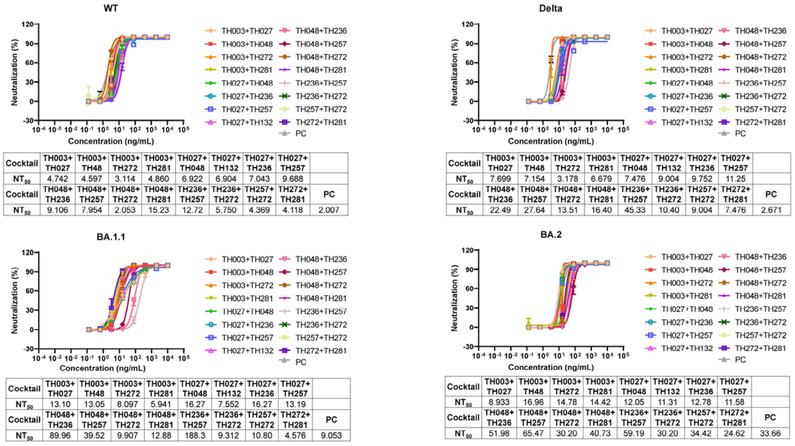

c

NT_{50} (ng/mL)	TH003+TH027	TH003+TH048	TH003+TH272	TH003+TH281	TH027+TH048	TH027+TH236	TH027+TH257	TH027+TH132	
WT	4.742	4.597	3.114	4.860	6.922	6.820	9.688	6.904	
Delta	7.699	7.154	3.178	6.679	7.476	9.752	11.25	9.004	
BA.1.1	13.10	13.05	8.097	5.941	16.27	61.86	13.19	7.552	
BA.2	8.933	16.96	14.78	14.42	12.05	12.78	11.58	11.31	
NT_{50} (ng/mL)	TH048+TH236	TH048+TH257	TH048+TH272	TH048+TH281	TH236+TH257	TH236+TH272	TH257+TH272	TH272+TH281	PC
WT	9.106	7.954	2.053	15.23	12.72	5.750	4.369	4.118	2.007
Delta	22.49	27.64	13.51	16.40	45.33	10.40	9.004	7.476	2.671
BA.1.1	89.96	39.52	9.907	12.88	188.3	9.312	10.80	4.576	9.053
BA.2	51.98	65.47	30.20	40.73	59.19	30.20	34.42	24.62	33.66

<25 | 25~50 | 50~100 | >100

Fig. S10. Neutralization potency of combinations of monoclonal antibodies against 4 SARS-CoV-2 strains.

(a) Mapping of antibody epitopes on the sequence alignment of selected VOCs. The mutation sites of the WT strain are shown in red. The epitopes of hACE2 are indicated by asterisks. The epitope residues of TH027 are shown in deep blue boxes. The epitope residues of TH132 are shown in orange boxes.

(b) Fitted curve of 16 paired antibodies against different SARS-CoV-2 strains by GraphPad Prism 8.0. PC as a positive control. (n=3 technical replicates). Data are presented as mean values ± SD.

(c) Neutralization potency of 16 combinatorial antibodies against different SARS-CoV-2 strains. PC as positive control.

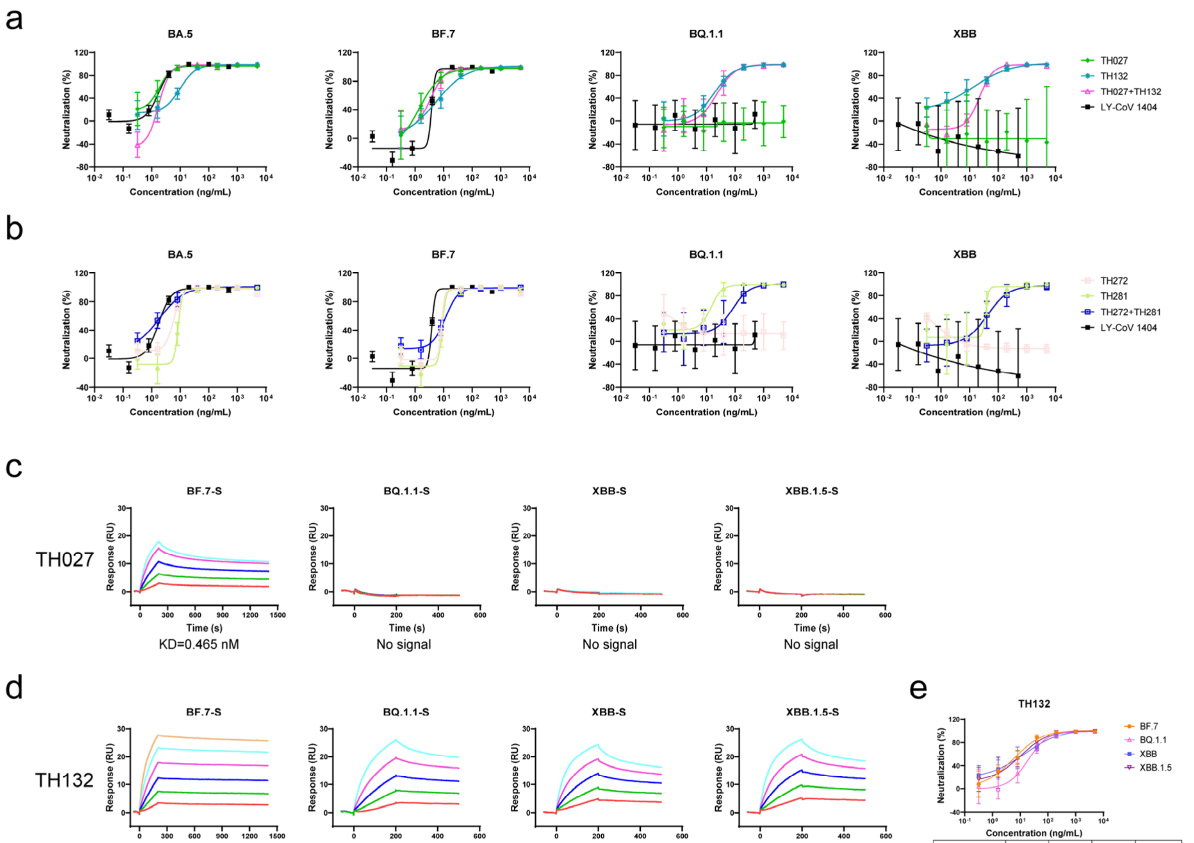

	BF.7	BQ.1.1	XBB	XBB.1.5
NT_{50} (ng/mL)	5.578	18.92	13.82	9.939

Fig. S11. Evaluation of elite antibodies against Omicron sublineages BQ and XBB.

(a) Fitted curve of TH027, TH132 and TH027+TH132 cocktail against BA.5, BF.7, BQ.1.1 and XBB by GraphPad Prism 8.0. (n=8 technical replicates). Data are presented as mean values ± SD.

(b) Fitted curve of TH272, TH281 and TH272+TH281 cocktail. LY-CoV1404 as control. (n=8). Data are presented as mean values ± SD.

(c) The affinity of TH027 to different Omicron sublineages was detected by SPR. Kinetic data from one representative experiment were fit to a 1:1 binding model by Biacore 8K. No signal means not detected.

(d) The affinity of TH132 to different Omicron sublineages was detected by SPR. Kinetic data from one representative experiment were fit to a 1:1 binding model by Biacore 8K. No signal means not detected.

(e) Fitted curve of TH132 against Omicron sublineages BF.7, BQ.1.1, XBB, and XBB.1.5 by GraphPad Prism 8.0. (n=8 technical replicates). Data are presented as mean values ± SD.

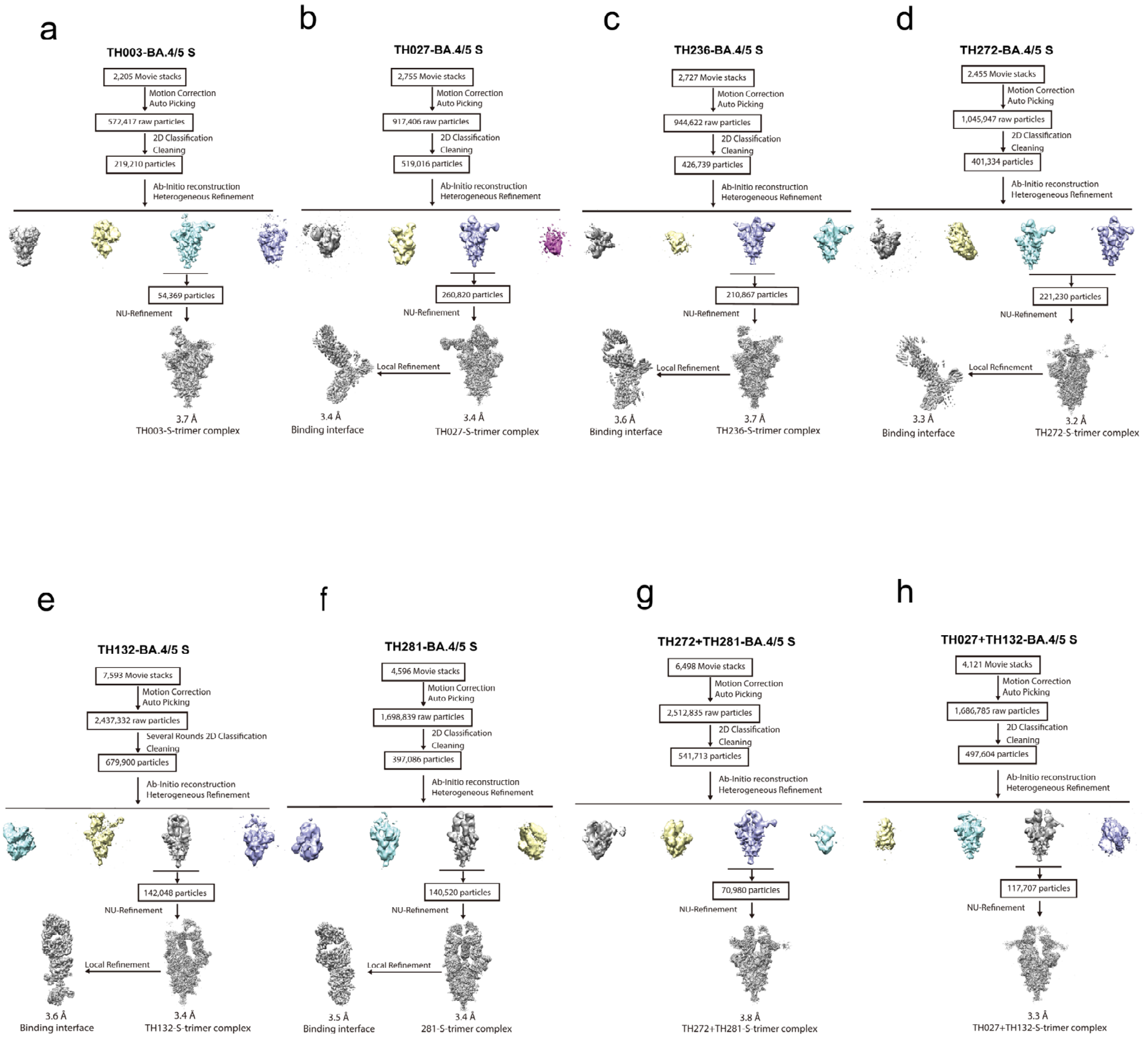

Fig. S12. Cryo-EM data processing flowcharts. Flowcharts for S protein in complex with Fab. (a) TH003, (b) TH027, (c) TH236, (d) TH272, (e) TH132, (f) TH281, (g) TH272+TH281, (h) TH027+TH132 are shown.

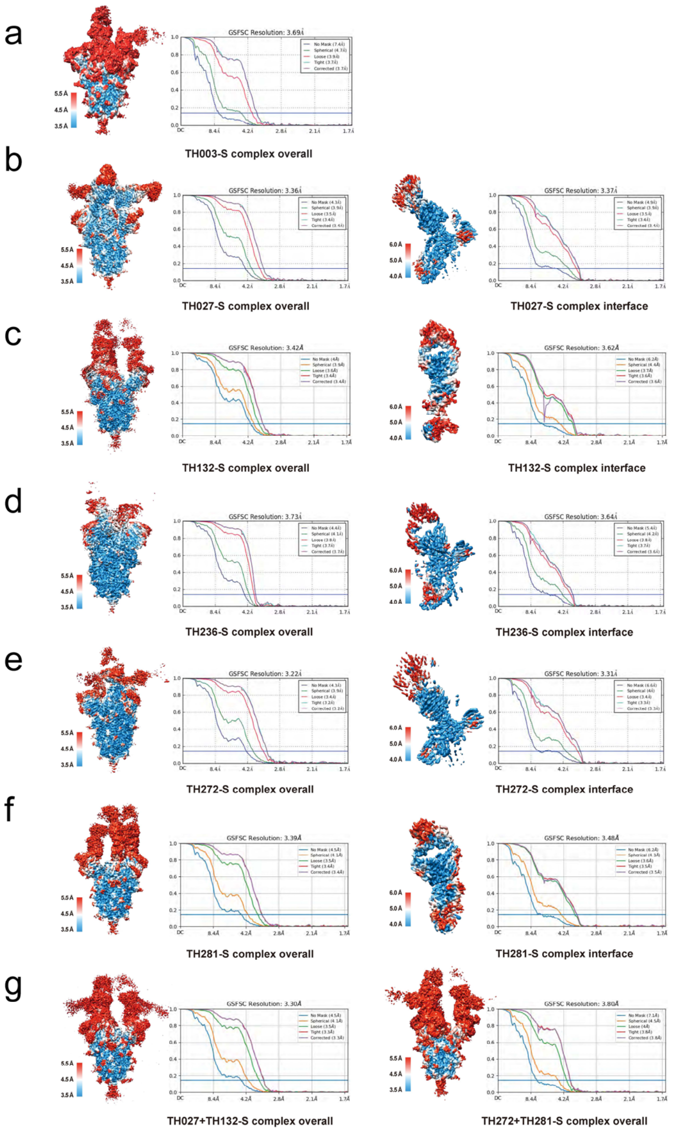

Fig. S13. Resolution estimation of the EM maps. Local resolution assessments of cryo-EM maps and the gold-standard FSC curves of overall maps of the S trimer in complex with Fabs and local maps of interfaces. (a) TH003, (b) TH027, (c) TH132, (d) TH236, (e) TH272, (f) TH281, (g) TH027+TH132 and TH272+TH281.

KD(M) by SPR									
Candidate antibodies	Spike (BA.1)	Spike (BA.4/5)	RBD (BA.1)	RBD (BA.2)	RBD (BA.5)	Spike (BF.7)	Spike (BQ.1.1)	Spike (XBB)	Spike (XBB.1.5)
TH003	2.55E-09	1.59E-09	1.29E-09	6.32E-10	4.84E-10				
TH004	8.27E-09	2.21E-09	3.19E-09	6.45E-10	1.56E-09				
TH015	5.98E-09	3.40E-09	5.00E-09	8.14EE-10	1.48E-09				
TH025	6.51E-09	2.94E-09	6.12E-09	4.90E-10	1.19E-09				
TH027	7.06E-09	1.86E-09	8.38E-10	9.40E-10	7.52E-10	4.65E-10	No signal	No signal	No signal
TH048	6.74E-10	No signal	8.39E-10	1.31E-08	No signal				
TH051	7.69E-09	6.79E-09	8.44E-09	1.44E-09	1.45E-09				
TH089	9.06E-09	8.96E-09	5.91E-09	1.92E-09	7.56E-10				
TH095	2.74E-09	1.54E-09	3.36E-09	8.30E-10	3.68E-10				
TH111	8.45E-09	5.38E-09	3.74E-09	1.10E-09	8.93E-10				
TH132	1.26E-09	1.71E-12	7.26E-10	1.71E-09	4.84E-10	9.12E-11	1.28E-09	1.34E-09	1.09E-09
TH139	4.55E-10	No signal	3.58E-10	5.56E-10	No signal				
TH183	2.14E-09	2.14E-09	1.34E-09	3.39E-10	1.41E-10				
TH236	8.67E-11	6.02E-10	3.00E-10	2.40E-09	6.21E-10				
TH257	6.50E-11	1.99E-10	1.02E-11	6.48E-11	5.89E-10				
TH272	1.33E-09	9.76E-13	2.96E-10	8.10E-12	4.50E-11				
TH273	2.63E-08	5.84E-12	2.12E-09	1.10E-12	1.79E-12				
TH274	8.75E-08	5.23E-11	4.57E-11	1.31E-12	6.71E-10				
TH281	5.17E-10	1.99E-10	1.19E-10	1.19E-10	7.81E-12				

Table S1. Summary of binding affinities measured by the SPR assay. The 19 candidate antibodies with S or RBD protein of Omicron's BA.1, BA.2, BA.4/5, BF.7, BQ.1.1, XBB, and XBB.1.5 variants were measured by the SPR assay.

NAbs		Total surface area, Å²		Interaction residues		Interface area, Å²	ΔiG (kcal/m)	ΔiG (P-value)	N_{HB}	N_{SB}	N_{DS}
		RBD	HC/LC	RBD	HC/LC						
TH003	HC	9458	6086	12	9	448.3	-4.2	0.348	6	0	0
	LC		5512	5	5	221.2	-0.3	0.651	3	2	0
TH027	HC	10426	6570	11	16	641.5	-2.6	0.721	9	3	0
	LC		6043	5	3	208.1	-3.5	0.238	0	2	0
TH132	HC	10756	6158	12	12	646.2	-4.9	0.562	10	0	0
	LC		5979	4	4	196.2	-1.9	0.522	2	0	0
TH236	HC	10681	7006	12	15	723.4	0.1	0.643	7	1	0
	LC		5801	4	8	264.8	0.5	0.820	4	0	0
TH272	HC	10333	6450	8	9	411.6	-2.7	0.581	2	2	0
	LC		5886	6	8	323.2	-3.7	0.355	5	0	0
TH281	HC	10836	6159	13	11	648.6	-5.4	0.494	8	0	0
	LC		5890	5	4	207.4	-2.0	0.519	3	0	0

HC: Heavy chain; LC: Light Chain; ΔiG: Solvation free energy gain upon formation of the interface; N_{HB}: number of potential hydrogen bonds across the interface; N_{SB}: number of potential salt bridges across the interface; N_{DS}: number of potential disulfide bonds across the interface

Table S2. PISA analysis of interaction between Fab_BA.4/5 RBD.

RBD	TH003	TH027	TH236	TH272	TH132	TH281
E340			Y105			
N343			S107			
T345		I30, S32	D106, S108, Y110			
R346	D31	L29, I30, W55	Y33, E99, P101, G103, Y104 Y110			
F347			Y104			
A348			Y104			
N354			Y105			
K356			Y105			
R403					(*N92*)	(N92)
T415					S56, F58	S56, F58
G416					F52, F58	F52,F58
N417					Y33, (*P95*)	Y33, (*P95*)
Y421					Y33, F52, A53,G54	Y33, F52, A53, G54
N439	W105, (*Y34*)	N105, T106, P107		(*Y34*)		
K440	A103,(*D52*)	H104, P107	(*G31, Y32*)	G33		
L441		S32, G34		W55		
S443	D104, W105	P107	(*S95*)	I102		
K444	D31, Y32, D104	Y54, W55, D56, D58, R60	(*Y93, T94, S95, S96, L99*)	Y54, D56, D58		
V445	A33, Y59, D104, (*Y93*)	Y54, Y109, (*W92*)	(*S96, S97*)	L52,Y54,R60,K100		
G446	S57, Y59	(*W92*)				
G447	Y53	R60		R60		
N448	Y53		Y59			
Y449	Y53		Y56, A57			
N450	Y53	D56	N54	D58		
R452			Y56			
L455					Y33	Y33
F456					V102	L99, V102
R457						A53
K458					A53	A53
N460					G54	G54
Y473					R31, A53	R31
Q474					R31	R31
A475					T28	T28, R31
Y489					R97, L99, V102	R97, L99
R498		(*D94*)		(*T95*)		
P499	W105, (*Y34*), (*Y93*)	(*F33*)		I102, (*Y34*), (*Y93*)		
T500	(*Y32*), (*G95*)	(*F33*)		(*V29*), (*G30*), (*A31*), (*T94*) (*A31*)		
Y501					(*S30*)	(*S30*)
G502						(*S28*)
H505					(*N92*)	(*N92*)
Q506				(*Q33*)		
R509		S32				

Distance <4 Å is used as the cutoff for contact.

Light chain residues are listed in italics in square braclets

Table S3. List of residues interacting between neutralizing antibodies and BA.4/5 RBD.

TH003-BA.4/5 RBD (PDB ID: 8GPY)	
Wavelength	0.9785
Resolution range	50.00 - 2.90 (2.75 - 2.69)
Space group	C 1 2 1
Unit cell	122.863 92.092 89.270 90.000 92.400 90.000
Total reflections	1265618
Unique reflections	29908 (954)
Redundancy	6.7
Completeness (%)	99.6 (89.2)
Mean I/sigma(I)	29.6 (3.0)
Wilson B-factor	37.54
R-merge	0.125 (0.882)
R-meas	0.136 (0.955)
R-pim	0.053 (0.363)
CC1/2	0.984 (0.814)
Reflections used in refinement	29905 (954)
Reflections used for R-free	2003 (62)
R-work	0.2172 (0.4219)
R-free	0.2550 (0.4712)
Number of non-hydrogen atoms	658
macromolecules	6409
solvent	149
Protein residues	828
RMS(bonds)	0.005
RMS(angles)	0.94
Ramachandran favored (%)	95.93
Ramachandran allowed (%)	3.95
Ramachandran outliers (%)	0.12
Rotamer outliers (%)	5.10
Clashscore	8
Average B-factor	44.96
macromolecules	45.20
solvent	34.56

Statistics for the highest-resolution shell are shown in parentheses.

Table S4. Crystallographic statistics.

	BA.4/5 spike trimer with TH003	BA.4/5 spike trimer with TH027	BA.4/5 spike trimer with TH132	BA.4/5 spike trimer with TH236	BA.4/5 spike trimer with TH272	BA.4/5 spike trimer with TH281	BA.4/5 spike trimer with TH27 + TH132	BA.4/5 spike trimer with TH272 +TH281
**PDB entry**	8GOU	7YVE	7YVG	7YVI	7YVK	7YVN	7YVO	7YVP
**EMDB entry**	EMD-34181	EMD-34124	EMD-34126	EMD-34128	EMD-34130	EMD-34133	EMD-34134	EMD-34135
**Data collection and processing**								
Magnification	29,000	29,000	29,000	29,000	29,000	29,000	29,000	29,000
Voltage (keV)	300	300	300	300	300	300	300	300
Electron exposure ($e^-/Å^2$)	50	50	50	50	50	50	50	50
Defocus range (μm)	1.2 - 2.4	1.2 - 2.4	1.2 - 2.4	1.2 - 2.4	1.2 - 2.4	1.2 - 2.4	1.2 - 2.4	1.2 - 2.4
Pixel size (Å)	0.82	0.82	0.82	0.82	0.82	0.82	0.82	0.82
Symmetry imposed	C1	C1	C1	C1	C1	C1	C1	C1
Initial particle images (no.)	572,417	917,406	2,437,332	944,622	1,045,947	1,608,839	1,686,785	2,512,835
Final particle images (no.)	54,369	260,820	142,048	210,867	221,230	140,520	117,707	70,980
Map global resolution (Å)	3.7	3.4	3.4	3.7	3.2	3.4	3.3	3.8
Global resolution FSC threshold	0.143	0.143	0.143	0.143	0.143	0.143	0.143	0.143
Map local resolution range (Å)	3.7 to 60	3.4 to 60	3.4 to 60	3.7 to 60	3.2 to 60	3.4 to 60	3.3 to 60	3.8 to 60
**Refinement**								
Model resolution (Å)	3.7	3.4	3.4	3.7	3.2	3.4	3.3	3.8
FSC threshold	0.143	0.143	0.143	0.143	0.143	0.143	0.143	0.143
Model resolution range (Å)	3.7 to 60	3.4 to 60	3.4 to 60	3.7 to 60	3.2 to 60	3.4 to 60	3.3 to 60	3.8 to 60
Map sharpening *B* factor ($Å^2$)	80.3	101.5	114.3	125.5	90.5	87.1	88.1	87.7
**Model composition**								
Non-hydrogen atoms	27,108	29,009	28,479	28,961	28,809	28,503	30,285	30,177
Protein residues	3,528	3,768	3,753	3,777	3,750	3,753	3,953	3,941
Ligands	37	37	37	37	37	37	37	37
***B* factors ($Å^2$)**								
Protein	210.84	113.10	88.69	178.48	119.17	170.57	177.65	211.66
Ligand	190.58	119.46	91,84	147.63	106.95	120.00	148.98	177.39
**R.m.s. deviations**								
Bond lengths (Å)	0.009	0.017	0.017	0.026	0.017	0.007	0.020	0.009
Bond angles (°)	1.220	1.453	1.545	1.862	1.522	1.246	1.613	1.275
**Validation**								
MolProbity score	2.13	2.07	2.08	2.27	2.06	2.02	2.34	2.18
Clashscore	13.4	11.20	14.89	18.98	12.39	12.18	22.69	16.39
**Ramachandran plot**								
Outliers	0.49	0.35	0.46	0.62	0.33	0.62	0.39	0.49
Allowed	4.67	5.04	5.52	5.33	5.47	5.28	5.56	5.45
Favored	94.84	94.61	94.02	94.05	94.20	94.10	94.05	94.06

Table S5. Cryo-EM Statistics for data collection, image processing and model building. (mAb-Spike trimer).

	TH27-RBD-interface	TH132-RBD-interface	TH236-RBD-interface	TH272-RBD-interface	TH281-RBD-interface
**PDB entry**	7YVF	7YVH	7YVJ	7YVL	7YVM
**EMDB entry**	EMD-34125	EMD-34127	EMD-34129	EMD-34131	EMD-34132
**Data collection and processing**					
Magnification	29,000	29,000	29,000	29,000	29,000
Voltage (keV)	300	300	300	300	300
Electron exposure ($e^{-}/Å^2$)	50	50	50	50	50
Defocus range (μm)	1.2 - 2.4	1.2 - 2.4	1.2 - 2.4	1.2 - 2.4	1.2 - 2.4
Pixel size (Å)	0.82	0.82	0.82	0.82	0.82
Symmetry imposed	C1	C1	C1	C1	C1
Initial particle images (no.)	917,406	2,437,332	944,622	1,045,947	1,608,839
Final particle images (no.)	260,820	142,048	210,867	221,230	140,520
Map global resolution (Å)	3.4	3.6	3.6	3.3	3.5
Global resolution FSC threshold	0.143	0.143	0.143	0.143	0.143
Map local resolution range (Å)	3.4 to 60	3.6 to 60	3.6 to 60	3.3 to 60	3.5 to 60
**Refinement**					
Model resolution (Å)	3.4	3.6	3.6	3.3	3.5
FSC threshold	0.143	0.143	0.143	0.143	0.143
Model resolution range (Å)	3.4 to 60	3.6 to 60	3.6 to 60	3.3 to 60	3.5 to 60
Map sharpening *B* factor ($Å^2$)	72.4	100.7	78.3	70.5	97.4
**Model composition**					
Non-hydrogen atoms	3,283	3,124	3,267	3,222	3,126
Protein residues	425	420	428	419	420
***B* factors ($Å^2$)**					
Protein	69.86	81.91	101.53	62.90	56.98
**R.m.s. deviations**					
Bond lengths (Å)	0.006	0.007	0.008	0.011	0.010
Bond angles (°)	1.080	1.310	1.199	1.334	1.484
**Validation**					
MolProbity score	1.94	2.36	2.60	2.41	2.61
Clashscore	8.26	15.22	24.91	18.35	27.76
**Ramachandran plot**					
Outliers	0.48	0.48	1.18	0.48	0.48
Allowed	7.64	8.70	8.29	8.96	8.94
Favored	91.89	90.82	90.52	90.56	90.58

Table S6. Cryo-EM Statistics for data collection, image processing and model building. (mAb-RBD interface).

DonorID	Sex	Age range	Severity	Vaccination
Donor1	male	31-40	moderate	vaccinated with 2 dose
Donor2	male	31-40	moderate	vaccinated with 2 dose
Donor3	male	51-60	moderate	unvaccinated
Donor4	male	21-30	mild	vaccinated with 2 dose
Donor5	female	31-40	moderate	vaccinated with 2 dose
Donor6	female	61-70	moderate	vaccinated with 1 dose
Donor7	female	61-70	moderate	vaccinated with 3 dose
Donor8	male	31-40	moderate	vaccinated with 3 dose
Donor9	male	51-60	moderate	vaccinated with 2 dose
Donor10	female	51-60	moderate	vaccinated with 3 dose
Donor11	male	31-40	mild	vaccinated with 2 dose
Donor12	female	31-40	moderate	vaccinated with 2 dose
Donor13	male	21-30	moderate	vaccinated with 3 dose
Donor14	female	31-40	moderate	vaccinated with 3 dose
Donor15	female	31-40	moderate	vaccinated with 2 dose
Donor16	female	21-30	mild	vaccinated with 3 dose
Donor17	female	31-40	moderate	vaccinated with 2 dose
Donor18	female	31-40	moderate	unvaccinated
Donor19	male	51-60	moderate	unvaccinated
Donor20	female	51-60	moderate	vaccinated with 2 dose
Donor21	female	31-40	moderate	vaccinated with 3 dose
Donor22	male	11-20	moderate	vaccinated with 2 dose
Donor23	male	41-50	moderate	vaccinated with 3 dose
Donor24	female	21-30	mild	vaccinated with 3 dose
Donor25	female	51-60	moderate	vaccinated with 3 dose
Donor26	male	41-50	moderate	vaccinated with 3 dose
Donor27	female	31-40	moderate	vaccinated with 3 dose
Donor28	female	81-90	moderate	unvaccinated
Donor29	female	61-70	moderate	vaccinated with 3 dose
Donor30	male	51-60	moderate	vaccinated with 2 dose
Donor31	male	31-40	moderate	NA
Donor32	male	51-60	moderate	vaccinated with 2 dose
Donor33	male	41-50	mild	NA
Donor34	female	51-60	moderate	NA
Donor35	female	51-60	moderate	NA
Donor36	male	21-30	mild	NA
Donor37	female	61-70	moderate	NA
Donor38	male	31-40	moderate	NA

Note: Except for donor1, dornor2, and donor3, the remaining donors were randomly divided into three groups (bulk1, bulk2, and bulk3).

Table S7. Donor information.
